# Nomograms for Predicting Cancer-Specific and Overall Survival Among Patients With Endometrial Carcinoma: A SEER Based Study

**DOI:** 10.3389/fonc.2020.00269

**Published:** 2020-03-19

**Authors:** Lingping Zhu, Xiaoming Sun, Wenpei Bai

**Affiliations:** ^1^Department of General Practice, Zhongshan Hospital, Fudan University, Shanghai, China; ^2^Health Development Research Centre of Pudong Institute for Health Development, Pudong, China; ^3^Department of Gynecology, Beijing Shijitan Hospital, Beijing, China

**Keywords:** endometrial carcinoma, nomograms, cancer-specific survival, overall survival, medical technology progress

## Abstract

**Background:** This study aimed to develop a detailed survival prognostication tool based on various clinical indicators of patients because of the lack of comprehensive prognostic tool.

**Methods:** Data regarding 63,729 patients with endometrial carcinoma were extracted from the SEER database between 1988 and 2015. Univariate and multivariate Cox regression analyses were used to screen for meaningful independent prognostic factors. These factors were used to construct a nomogram model, a survival prognostication tool for 3- and 5-year tumor-specific survival and overall survival among patients with endometrial carcinoma.

**Results:** A total of 63,729 patients were randomly assigned to the training group (*n* = 42,486) and the test group (*n* = 21,243). Age, race, year of diagnosis, histologic grade, clinical stage, and tumor size were assessed as predictors of cancer-specific survival (CSS) and univariate and multivariate Cox regression analyses were used to identify independent prognostic factors (*P* < 0.05). Finally, a nomogram was constructed, the predicted C-indices for cancer-specific survival and overall survival training groups were 0.859 (95% confidence interval 0.847–0.871) and 0.782 (95% confidence interval 0.772–0.792).

**Conclusions:** Nomograms constructed using various clinical indicators can provide better and more accurate predictions for patients with endometrial carcinoma. Those nomograms could help identify patients with high-risk endometrial carcinoma.

## Introduction

Endometrial carcinoma is a common epithelial malignancy of the endometrium in the female reproductive system worldwide. Its incidence rate is increasing while the age of onset is decreasing each year (1). The prognosis of endometrial carcinoma depends mainly on the stage of the disease and histological type. The 5-year survival rates of stages I and II endometrial carcinoma are about 80–90 and 70–80%, respectively, while that of stage III and IV disease is 20–60% (2, 3). Some previous studies used biomarkers to estimate the prognosis of endometrial carcinoma. A model used a combination of 7-LncRNA and clinical indicators to predict the survival prognosis of endometrial carcinoma with a C-index of 0.801. Four indicators, including clinical stage, age, pathological grade, and LncRNA were included in the nomogram of prediction; however, the sample size was small (4). At the same time, there is no accurate estimation of the survival rate of patients with endometrial carcinoma brought about by advancement in medical technology. The evaluation of the improvement of the survival rate due to medical technology among patients with endometrial carcinoma is a subject worthy of further study. The present study has the characteristics of small sample size, insufficient analysis factors, and insufficient data.

The linked Surveillance, Epidemiology, and End Results (SEER) database is a large population-based source of information for all cancer-related epidemiologic and health-related services research. It captures data regarding nearly 25% of the US population, and contains all kinds of clinical information, social information, and cost of treatment, all of which freely provide researchers with enough information (5). In this study, we assessed endometrial carcinoma patients registered between 1988 and 2015 in the SEER database, and aimed to develop validated prognostic nomograms regarding overall survival (OS) and cancer-specific survival (CSS) to estimate the change in the extent of surveillance from the medical technological progress for endometrial carcinoma patients.

## Materials and Methods

### Patient Enrollment and Variables

All patients' information was obtained from the SEER database using the SEER^*^Stat software (version 8.3.5; National Cancer Institute, USA).

The time limit for data collection was from 1988 to 2015. The inclusion criteria were as follows: (1) diagnosis of endometrial carcinoma (International Classification of Diseases for Oncology: 8380/3, histology: 8140-8389) and (2) known cause of death and survival duration after diagnosis.

The exclusion criteria were as follows: (1) unknown use of radiotherapy or chemotherapy, (2) unknown diagnostic method, (3) unknown histology grade classification, (4) unknown clinical stage, (5) unknown exact tumor size, (6) unknown metastasis information, and (7) unknown race information of patient.

**Figure 1:**
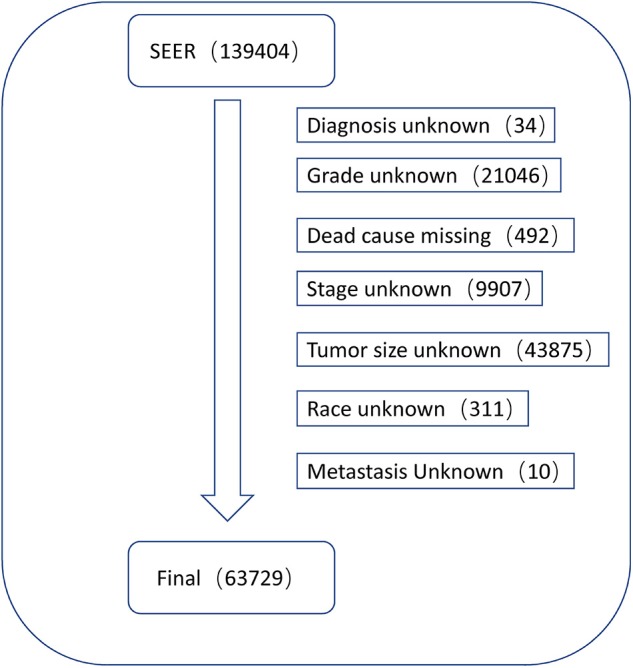
The flow chart of data process.

**Figure 2:**
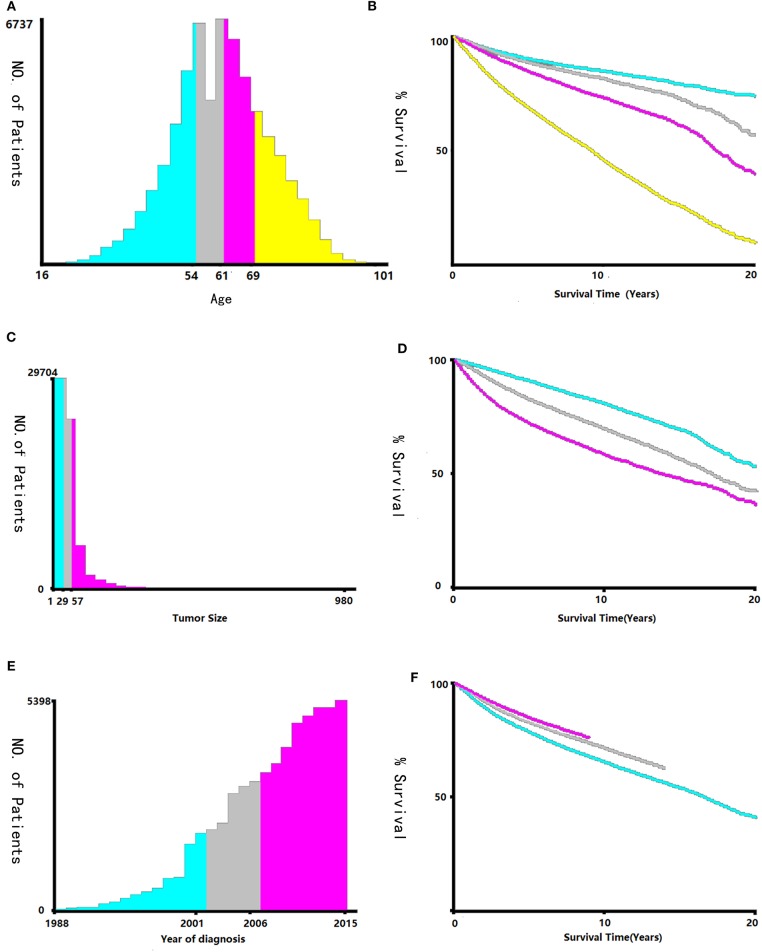
Identification of optimal cut-off values of age **(A,B)**, tumor size **(C,D)**, and year of diagnosis **(E,F)** via X-tile software analysis. Optimal cut-off values of age were identified as 54, 61 and 69-years based on overall survival. Optimal cut-off values of tumor size were identified as 29 mm and 57 mm based on overall survival. Optimal cut-off values of year of diagnosis were identified as 2001 and 2006 based on overall survival.

**Figure 3:**
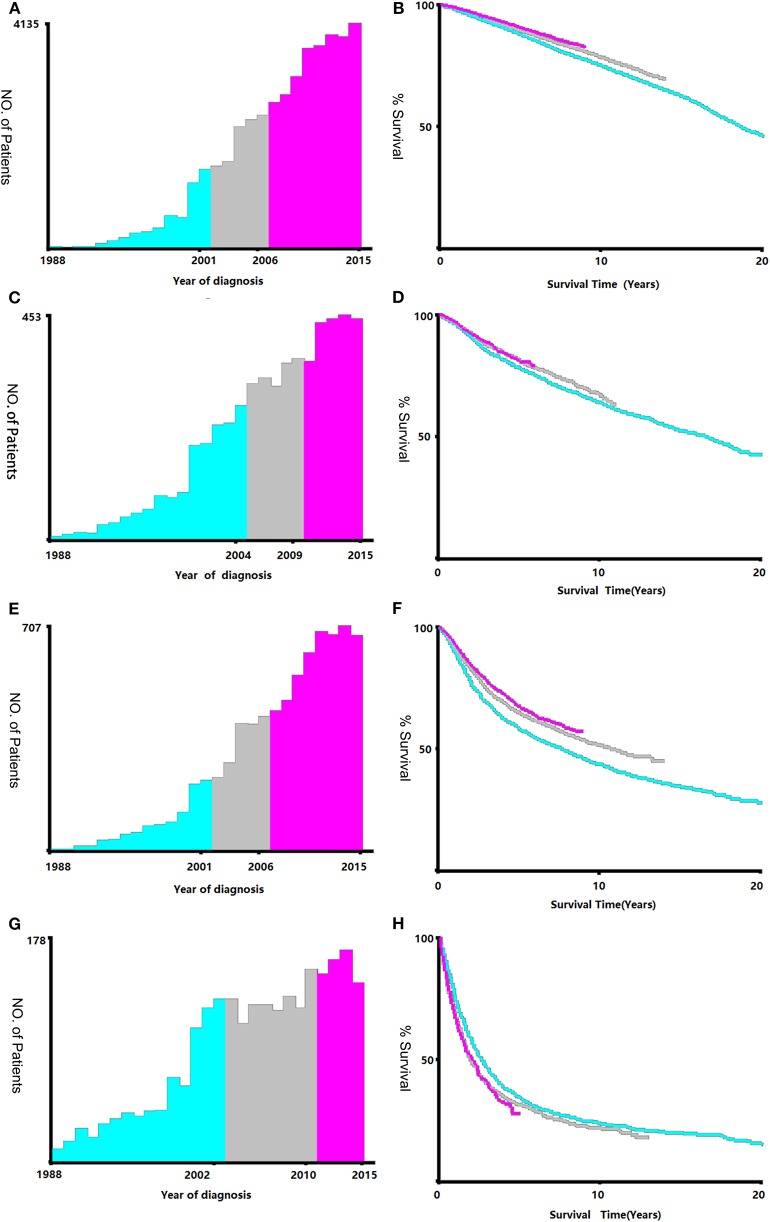
Identification of optimal cut-off values of year of diagnosis [Stage I **(A,B)**, Stage II **(C,D)**, Stage III **(E,F)**, Stage IV **(G,H)**]. Optimal cut-off values of year of diagnosis in stage I were identified as 2001 and 2006 based on overall survival. Optimal cut-off values of year of diagnosis in stage II were identified as 2004 and 2009 based on overall survival. Optimal cut-off values of year of diagnosis in stage III were identified as 2001 and 2006 based on overall survival. Optimal cut-off values of year of diagnosis in stage IV were identified as 2002 and 2010 based on overall survival.

Data regarding clinical characteristics including age, race, histologic grade, clinical stage, year of diagnosis, tumor size, metastatic status, tissue region, radiation, chemotherapy, survival time, cause of death, and survival status were collected from the SEER database. One lakh thirty nine thousand four hundred and four patients were collected from the SEER database, the data process flowchart was presented in Figure 1. X-tile software (Yale University, New Haven, Connecticut, USA) was used to assess the optimal cut-off values for age, tumor size, and year of diagnosis (Figure 2). The optimal cut-off values for age were 54-, 61-, and 69-years; the optimal cut-off values for tumor size were 29 and 57 mm; the optimal cut-off values for the year of diagnosis were 2001 and 2006. We also used X-tile software to assess the best cut-off for year of diagnosis according to different clinical stages (Figure 3), described as localized, regional, and distant in accordance with the American Joint Committee on Cancer criteria. Radiation status was classified as with radiation and without radiation. Chemotherapy status was classified as with chemotherapy and without chemotherapy.

According to the inclusion and exclusion criteria, 63,729 patients with endometrial carcinoma were finally enrolled in our study, all of whom were randomly divided into the training cohort (*n* = 42,486) and test cohort (*n* = 21,243) in the ratio 7:3.

The endpoints of our study included CSS and OS; however, the 3- and 5-year survival of both endpoints were used as well.

### Statistical Analysis

Continuous and categorical data are expressed as frequencies with percentages. A chi-square test was performed to explore the relationship between the clinical features of the two groups. The optimal cut-off values of age, tumor size, and year of diagnosis were assessed using the X-tile software as previously mentioned (Figure 2). Analysis items with *P* < 0.05 were considered statistically significant. Univariate and multiple Cox regression analysis was used to assess independent survival-related factors among our clinical data. The chi-square test and Cox regression analysis were performed using SPSS version 22.0 (IBM Corp., Armonk, NY, USA). Meanwhile, 95% confidence intervals (CIs) and hazard ratios were calculated. The nomograms according to both cohorts were validated both internally and externally. The C-index (Harrell's concordance index) was used to assess the exact prognostic values of nomograms. The verification curve in this study could show the consistency between the actual and predicted nomograms. Nomograms and the verification curve were constructed and adjusted using R version 1.1.453 (The R Development Core Team, Vienna, Austria) in the RStudio environment.

## Results

### Patients' Basic Information

A total of 63,729 out of 139,404 patients registered with endometrial carcinoma between 1988 and 2015 were enrolled from the SEER database according to the inclusion and exclusion criteria. The patients were divided into the training group (*n* = 42,486) and test group (*n* = 21,243). The basic information of the patients is listed in Table 1. There were 53,583 (84.1%) White people, 4,006 (6.3%) black people, and 6,140 (9.6%) people of other races (American Indians/Alaska Natives, and Asian/Pacific Islanders). In all 7,654 (12.0%) people were diagnosed before 2002, 13,809 (21.7%) people were diagnosed between 2002 and 2006, and 42,266 (66.3%) people were diagnosed after 2007. The numbers of people with histologic grades G1, G2, G3, and Gx were 29,028 (45.5%), 22,722 (35.7%), 10,577 (16.6%), and 1,422 (2.2%), respectively. The numbers of people with clinical stages I, II, III, and IV were 47,055 (73.8%), 5,877 (9.2%), 8,212 (12.9%), and 2,585 (4.1%); those with metastatic status M0 and M1 were, respectively, 61,376 (96.3%) and 1,577 (3.7%); and those with localized, regional, and distant surgical stage were, respectively, 46,685 (73.3%), 11,878 (18.6%), and 5,166 (8.1%). The number of patients with and without radiation were, respectively, 48,333 (75.8%) and 15,396 (24.2%); the number of those with and without chemotherapy were, respectively, 53,571 (84.1%) and 10,158 (15.9%); and those with tumor diameter ≤ 28, 29–56, and ≥57 mm were, respectively, 23,219 (36.4%), 26,229 (41.2%), and 14,281(22.4%). The number of patients aged ≤ 54-, 55–61-, 62–69-, and ≥70-years were 17,620 (27.6%), 15,648 (24.6%), 15,001 (23.5%), and 15,460 (24.3%) (Table 1). The chi-test for all of those variables between the two groups yielded *P* > 0.05. In the univariate Cox regression analysis for CSS, except for age between 55- and 61-years, other variables were significantly meaningful (*P* < 0.05). In the univariate Cox regression for OS, all variables were significant meaningful (*P* < 0.05) (Table 2). In the multivariate Cox regression for CSS, except for “other” in race, and regional in surgical stage, all of other variables were meaningful (*P* < 0.05). In the multivariate Cox regression for OS, except for regional and distant in surgical stages, all other variables were meaningful (*P* < 0.05) (Table 3).

**Table 1:** Baseline demographic and clinical characteristics of patients with endometrial carcinoma.

**Variables**		**Training Cohort (%)**		**Test Cohort (%)**		**Total (%)**	***P***
Race, *n*, %							0.718
White	35,750	84.1	17,833	83.9	53,583	84.1	
Black	2,648	6.2	1,358	6.4	4,006	6.3	
Other	4,088	9.6	2,052	9.7	6,140	9.6	
Year of Diagnosis, *n*, %							0.713
≤ 2001	5,131	12.1	2,523	11.9	7,654	12.0	
2002–2006	9,181	21.6	4,628	21.8	13,809	21.7	
≥2007	28,174	66.3	14,092	66.3	42,266	66.3	
Histology, *n*, %							0.741
G1	19,353	45.6	9,675	45.5	29,028	45.5	
G2	15,125	35.6	7,597	35.8	22,722	35.7	
G3	7,041	16.6	3,516	16.6	10,577	16.6	
Gx	967	2.3	455	2.1	1,422	2.2	
Clinical Stage, *n*, %							0.450
Stage I	31,364	73.8	15,691	73.9	47,055	73.8	
Stage II	3,873	9.1	2,004	9.4	5,877	9.2	
Stage III	5,512	13.0	2700	12.7	8,212	12.9	
Stage IV	1,737	4.1	848	4.0	2,585	4.1	
Metastasis, *n*, %							0.710
M0	40,909	96.3	20,467	96.3	61,376	96.3	
M1	1,577	3.7	776	3.7	2,353	3.7	
Surgical Stage, *n*, %							0.128
Localized	31,110	73.2	15,575	73.3	46,685	73.3	
Regional	7,871	18.5	4,007	18.9	11,878	18.6	
Distant	3,505	8.2	1,661	7.8	5,166	8.1	
Radiation, *n*, %							0.120
NO	32,300	76.0	16,033	75.5	48,333	75.8	
YES	10,186	24.0	5,210	24.5	15,396	24.2	
Chemotherapy, *n*, %							0.233
NO	35,662	83.9	17,909	84.3	53,571	84.1	
YES	6,824	16.1	3,334	15.7	10,158	15.9	
Tumor Size, *n*, %							0.854
≤ 28	15,510	36.5	7,709	36.3	23,219	36.4	
29–56	17,474	41.1	8,755	41.2	26,229	41.2	
≥57	9,502	22.4	4,779	22.5	14,281	22.4	
Age, *n*, %							0.293
≤ 54	11,699	27.5	5,921	27.9	17,620	27.6	
55–61	10,420	24.5	5,228	24.6	15,648	24.6	
62–69	9,964	23.5	5,037	23.7	15,001	23.5	
≥70	10,403	24.5	5,057	23.8	15,460	24.3	

*Gx means undifferentiated tissue cells*.

**Table 2:** Univariate cox regression analysis of cancer-specific survival and Overall survival in the training cohort.

**Variables**		**Cancer-specific survival**			**Overall survival**	
	**HR**	**95% CI**	***P***	**HR**	**95% CI**	***P***
**AGE**						
**≤54**	**Reference**			**Reference**		
55–61	1.074	0.973–1.185	0.159	1.270	1.178–1.369	<0.001^*^
62–69	1.404	1.276–1.545	<0.001^*^	1.917	1.786–2.056	<0.001^*^
≥70	2.922	2.680–3.184	<0.001^*^	4.590	4.316–4.881	<0.001^*^
**RACE**						
**White**	**Reference**			**Reference**		
Black	1.867	1.681–2.074	<0.001^*^	1.488	1.380–1.605	<0.001^*^
Other	0.865	0.770–0.972	0.014^*^	0.738	0.680–0.802	<0.001^*^
**YEAR OF DIAGNOSIS**						
**≤2001**	**Reference**			**Reference**		
2002–2006	0.554	0.507–0.605	<0.001^*^	0.765	0.723–0.810	<0.001^*^
≥2007	0.420	0.387–0.457	<0.001^*^	0.666	0.629–0.706	<0.001^*^
**HISTOLOGY**						
**G1**	**Reference**			**Reference**		
G2	3.247	2.936–3.592	<0.001^*^	1.723	1.634–1.817	<0.001^*^
G3	10.139	9.195–11.180	<0.001^*^	3.618	3.425–3.822	<0.001^*^
Gx	14.339	12.410–16.568	<0.001^*^	4.836	4.363–5.361	<0.001^*^
**CLINICAL STAGE**						
**Stage I**	**Reference**			**Reference**		
Stage II	3.118	2.791–3.484	<0.001^*^	1.736	1.621–1.858	<0.001^*^
Stage III	7.342	6.781–7.949	<0.001^*^	2.973	2.820–3.134	<0.001^*^
Stage IV	24.888	22.824–27.140	<0.001^*^	7.869	7.382–8.387	<0.001^*^
**SURGICAL STAGE**						
**Localized**	**Reference**			**Reference**		
Regional	4.736	4.370–5.132	<0.001^*^	2.193	2.086–2.306	<0.001^*^
Distant	14.836	13.724–16.039	<0.001^*^	4.969	4.711–5.242	<0.001^*^
**RADIATION**						
**NO**	**Reference**			**Reference**		
YES	1.537	1.436–1.646	<0.001^*^	1.230	1.174–1.288	<0.001^*^
**CHEMOTHERAPY**						
**NO**	**Reference**			**Reference**		
YES	3.753	3.516–4.006	<0.001^*^	1.978	1.833–2.077	<0.001^*^
**TUMOR SIZE**						
**≤28**	**Reference**			**Reference**		
29–56	2.424	2.201–2.669	<0.001^*^	1.713	1.625–1.806	<0.001^*^
≥57	6.015	5.478–6.605	<0.001^*^	2.713	2.567–2.868	<0.001^*^

*HR, Hazard Ratio; CI, Confidence Interval*.

* *means p <0.05, Gx means undifferentiated tissue cells*.

**Table 3:** Multivariate cox regression analysis of cancer-specific survival and Overall survival in the training cohort.

**Variables**		**Cancer-specific survival**			**Overall survival**	
	**HR**	**95% CI**	***P***	**HR**	**95% CI**	***P***
**AGE**						
**≤54**	**Reference**			**Reference**		
55–61	1.351	1.223–1.493	<0.001^*^	1.437	1.333–1.550	<0.001^*^
62–69	1.788	1.623–1.971	<0.001^*^	2.186	2.036–2.347	<0.001^*^
≥70	3.194	2.922–3.492	<0.001^*^	4.877	4.578–5.195	<0.001^*^
**RACE**						
**White**	**Reference**			**Reference**		
Black	1.506	1.354–1.675	<0.001^*^	1.421	1.317–1.534	<0.001^*^
Other	0.952	0.847–1.070	0.413	0.896	0.825–0.973	0.009^*^
**YEAR OF DIAGNOSIS**						
**≤2001**	**Reference**			**Reference**		
2002–2006	0.788	0.721–0.863	<0.001^*^	0.924	0.873–0.978	0.007^*^
≥2007	0.633	0.580–0.689	<0.001^*^	0.868	0.819–0.9121	<0.001^*^
**HISTOLOGY**						
**G1**	**Reference**			**Reference**		
G2	2.007	1.810–2.225	<0.001^*^	1.307	1.238–1.380	<0.001^*^
G3	3.712	3.338–4.128	<0.001^*^	1.993	1.876–2.117	<0.001^*^
Gx	4.268	3.663–4.974	<0.001^*^	2.349	2.109–2.616	<0.001^*^
**CLINICAL STAGE**						
**Stage I**	**Reference**			**Reference**		
Stage II	1.591	1.026–2.468	0.038^*^	1.382	1.033–1.849	<0.001^*^
Stage III	3.165	2.065–4.850	<0.001^*^	2.212	1.667–2.936	<0.001^*^
Stage IV	7.699	5.029–11.787	<0.001^*^	4.976	3.752–6.599	<0.001^*^
**SURGICAL STAGE**						
**Localized**	**Reference**			**Reference**		
Regional	1.425	0.923–2.200	0.110	1.111	0.833–1.480	0.475
Distant	1.668	1.088–2.558	0.019^*^	1.229	0.929–1.627	0.149
**RADIATION**						
**NO**	**Reference**			**Reference**		
YES	0.872	0.811–0.937	<0.001^*^	0.799	0.761–0.840	<0.001^*^
**CHEMOTHERAPY**						
**NO**	**Reference**			**Reference**		
YES	0.841	0.777–0.911	<0.001^*^	0.866	0.814–0.920	<0.001^*^
**TUMOR SIZE**						
**≤28**	**Reference**			**Reference**		
29–56	1.605	1.455–1.771	<0.001^*^	1.368	1.297–1.444	<0.001^*^
≥57	2.345	2.118–2.597	<0.001^*^	1.747	1.642–1.858	<0.001^*^

*HR, Hazard Ratio; CI, Confidence Interval*.

* *means p <0.05, Gx means undifferentiated tissue cells*.

### Comparison With Chemotherapy and Radiotherapy in Each Clinical Stage

**Figure 4:**
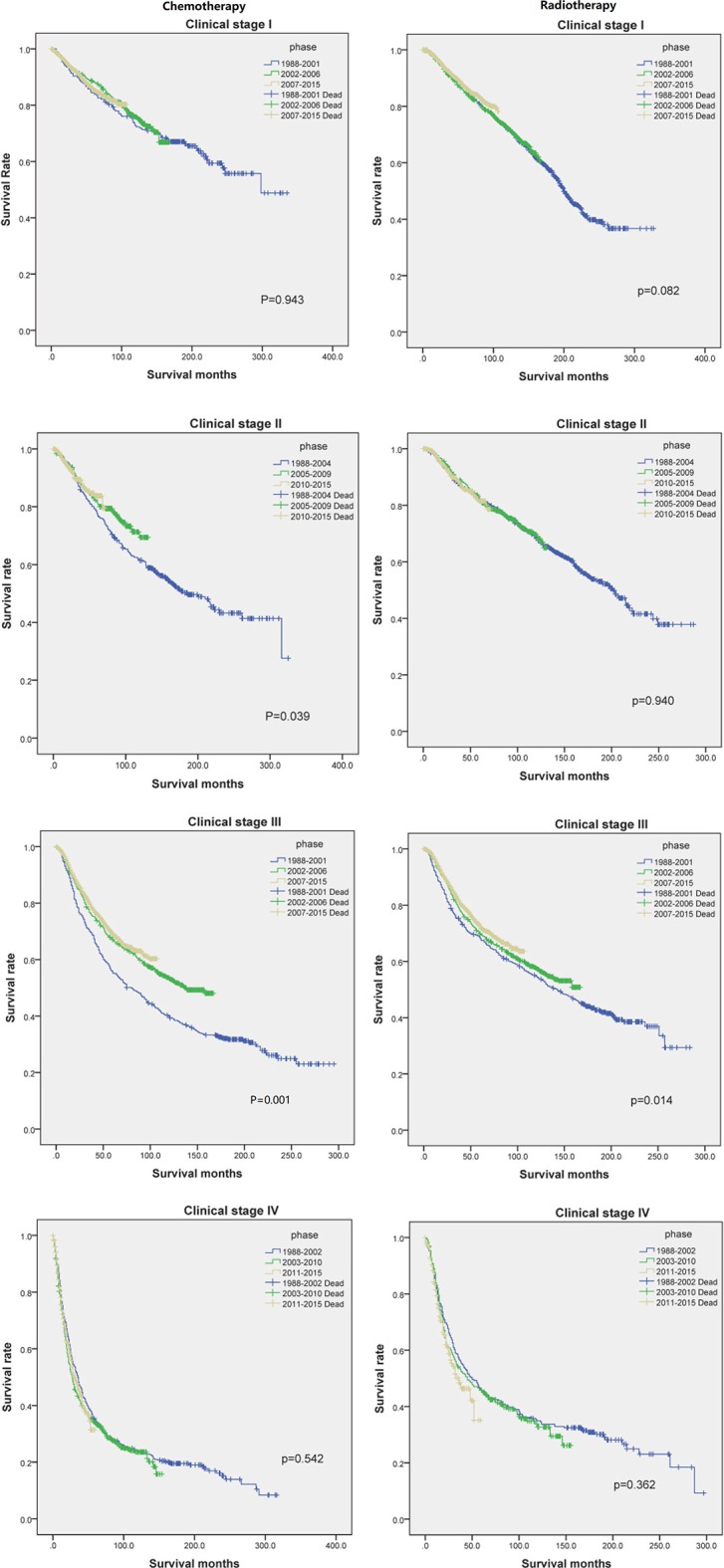
The Kaplan-Meier plot of each clinical stage compared with chemotherapy and radiotherapy in each phase.

Figure 4 shows us the Kaplan-Meier plot in each clinical stage compared with chemotherapy and radiotherapy, the right column shows chemotherapy treatment have made progress in clinical stage II and III, the left column shows radiotherapy treatment have made progress in clinical stage III, both treatment didn't show any progress in clinical stage I and IV, the treatment in clinical stage IV even showed worse effect than previous one.

### Construction and Validation of the Nomograms for OS and CSS

**Figure 5:**
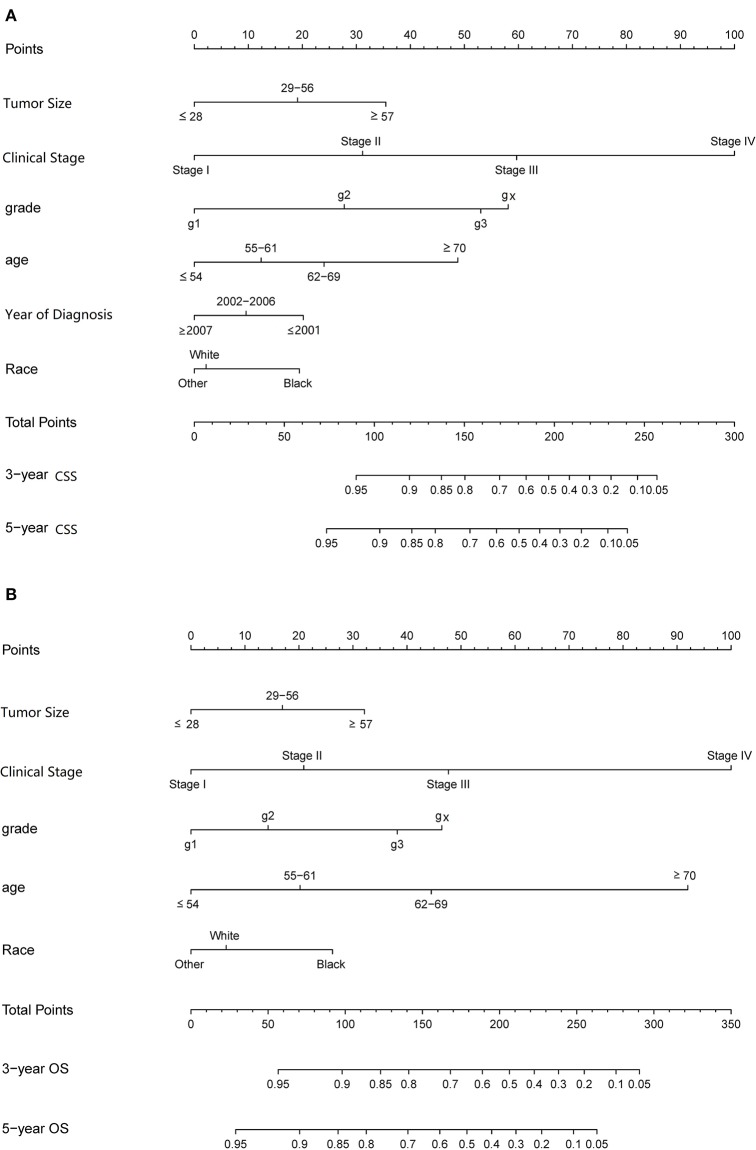
Nomograms to predict 3- and 5-year of cancer-specific survival **(A)** and overall survival **(B)** for patients with endometrial carcinoma.

**Figure 6:**
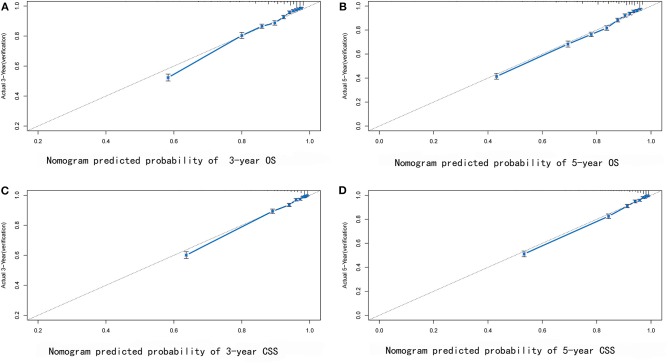
External verification plots of 3-year **(A)** and 5-year **(B)** overall survival nomogram verification curves; 3-year **(C)** and 5-year **(D)** cancer-specific survival nomogram verification curves.

Some non-significant variables or variables with light effects were excluded. Patient age, histologic grade, clinical stage, tumor size, year of diagnosis, and race were used to construct the nomogram for CSS (Figure 5). Patient age, histologic grade, clinical stage, tumor size, and race were used to construct the nomogram for OS (Figure 5). The exact point for each variable is listed in Table 4. We also validated the nomograms internally and externally. The C-index was used to assess the predictive accuracy of the nomograms. For the internal validation of the nomogram, the C-indexes were, respectively, 0.859 (0.847–0.871) and 0.782 (0.772–0.792) for CSS and OS. For the external validation of the nomogram, the C-indexes were, respectively, 0.859 (0.841–0.876) and 0.782 (0.766–0.798) for CSS and OS. The validation for both nomograms showed a good level of agreement on the prediction value (Figure 6).

**Table 4:** Scores of prognostic factors in the CSS and OS nomograms.

**Characteristic**	**OS nomogram**	**CSS nomogram**
**Age**		
≤ 54	0	0
55–61	20	12
62–69	44	24
≥70	92	49
**Size**		
≤ 28	0	0
29–56	17	19
≥57	32	35
**Race**		
White	7	2
Black	26	19
Other	0	0
**Clinical stage**		
Stage I	0	0
Stage II	21	31
Stage III	48	60
Stage IV	100	100
**Tissue grade**		
G1	0	0
G2	14	28
G3	38	53
Gx	46	58
**Year of diagnosis**		
≤ 2001		0
2002–2006		10
≥2007		20

*Gx means undifferentiated tissue cells*.

## Discussion

The survival prognosis of endometrial carcinoma has improved significantly with advances in medical technology; however, there are no studies showing the changes in the survival prognosis of endometrial carcinoma with advancements in medical technology. The X-tile software can provide optimal cut-off points for continuous variables affecting tumor survival prognosis (6). Using the X-tile software to assess the best cut-off point for the year of diagnosis showed that survival rate of endometrial carcinoma was a turning point in 2001. Because the screening methods for endometrial carcinoma have not changed basically, the improvement in survival rate was probably due to changes in staging (7); improvement of surgical methods, radiotherapy progress, and chemotherapy progress; comprehensive treatment of endometrial carcinoma (8); and other factors. There were fewer chemotherapy drugs before 2001, mainly chemotherapeutic drugs such as cisplatin and doxorubicin (9). These drugs were mainly used for advanced endometrial carcinoma; there are more choices of chemotherapeutic drugs than before (7) and hormone therapy has been used in the treatment of endometrial carcinoma since 2001 (10). Through our research, we found that chemotherapy drugs can improve the survival rate of patients in the early stage (clinical stage II and III) (Figure 4), but by extending the observation time, the previous chemotherapy drugs may increase the mortality rate among patients in clinical stage IV. By assessing the patients according to each clinical stage (Figure 3), we found that the survival rate of endometrial carcinoma was improved in patients with three preclinical stages, particularly for patients with clinical stage III, and the improvement in survival rate was the most obvious. In the clinical stage IV, the patient survival rate decreased. Due to the limitation of observation time, it was impossible to completely determine whether the final three curves would coincide. However, in the early stage after the diagnosis of stage IV, there was no advancement in medical technology that could result in a significant increase in survival rate. The optimal time cut-off points for different clinical stages are inconsistent, and the final reason may be due to the inconsistent benefits of medical technology for patients at different stages and at different time periods.

In this study, the X-tile software used the ages of 54, 61, and 69 as the cut-off points, which could better distinguish the survival rate, particularly in terms of OS. Even its role exceeds the effect of histology grade. At the same time, with the tumor diameters of 29 and 56 mm as cut-off values, it can also distinguish its survival rate better than before. Inclusion of these two indicators in our current tumor staging system may improve the current clinical staging system for endometrial carcinoma.

We have constructed a different prognostic model for assessing the CSS and OS of endometrial carcinoma. The CSS predictive model consists of age, clinical stage, pathological tissue grade, tumor size, ethnicity, and diagnostic time, while the OS predictive model consists of the first five items. It can be seen from these differences that different diagnostic times will lead to different survival rates among patients, which indirectly suggests the impact of advancement in medical technology on the survival prognosis of patients with endometrial carcinoma. At the same time, the other difference between the two models is the effect of age at diagnosis on the survival rate. For CSS, the effect of age at diagnosis was no greater than the effect of histopathological grade on survival prognosis; however, for OS, the effect of age at diagnosis was obviously greater than that of the latter. According to the FIGO 2009 staging, the 5-year survival rate of endometrial carcinoma in clinical stage I was about 77.6–89.6%, that of stage III was 73.5%, that of stage III is about 49.4–56.3%, and that of stage IV is about 21.1–22.0%, but the study failed to correct for tumor size and the impact of chemotherapy, and a prediction model was not constructed. Our results are similar with respect to age, histopathological grade, clinical stage, radiotherapy, and other factors on survival rate, although factors such as marriage and radiotherapy were not corrected in our study; however, the effects of these factors were not important. At the same time, our study showed that the effect of tumor size on nomogram was significant, and the chemotherapeutic factors were also corrected in the study. There was a nomogram for predicting the prognosis of endometrial carcinoma in 2010. Age, number of negative lymph nodes, clinical stage, histologic grade, and histologic type were used to construct this nomogram, and this model showed that the OS rate of patients with endometrial carcinoma was greater, and the effect was greater. The histologic type had the smallest effect, but due to the small number of patients, only the 3-year survival rate is calculated, the model was not stable, and the predicted performance C-index of the 3-year OS was only 0.746 ± 0.011 (11). An analysis of endometrial carcinoma in the SEER database from 1973 to 1987 has been performed previously. A total of 41,120 patients were enrolled according to the FIGO 1971 staging standard. The 5-year survival rates for clinical stages I, II, III, and IV were 86–93.9, 72.9, 48.1, and 25.4%, respectively. All of those are similar to the previous study results, but failed to correct for the effects of radiotherapy, chemotherapy, and tumor size, and failed to construct a model (12). Previous studies that used biomarkers to construct endometrial carcinoma models have included fewer patients, the prognostic factors included were not sufficient (4), and the use of biomarkers worsened the economic benefits. Other factors related to the survival rate for endometrial carcinoma patients are LVSI (lymph-vascular space invasion) and some co-morbidity, as for the LVSI, there are some controversies about it, a study showed us that LVSI does not significantly compromise the survival outcome of Chinese EEC patients (13). But in some studies, LVSI shows potential prognostic value about locoregional recurrences in EEC patients (14).

In this study, to make the model more refined, we removed factors such as metastasis, surgical stage, radiotherapy, and chemotherapy. Radiotherapy and chemotherapy are meaningful factors, but did not yield an obvious effect on the model. We excluded these factors to make the model more concise for clinical application. The C-index identified in this study could be stabilized at around 0.8, which means our nomograms have a higher accuracy for predicting survival than before.

The strength of this study is that the number of patients included was large, and the overall observation time was longer. We also assessed more influencing factors, used correct analytical methods, drew meaningful conclusions, and constructed a model with practical application value. The limitation of our study was the failure to include more of the possibly related factors into the model because of limitations of database, censor data and the ease of use, such as marital status, Lymph node status, LVSI, and the chemical biomarkers. As most of the patients were white, failure to include more non-whites may have some impact on the application of the model. There may have some selection bias because nearly half of patients wasn't included in our study. Meanwhile, we didn't develop a very accurate model for the convenient use in our clinical practice, for example, the detailed chemotherapy and radiotherapy protocol. At the same time, the data of our build model and calibration model are from the SEER database, which may have some impact on its application. In the future, it is necessary to use other databases for correction.

In conclusion, this study found that advancement in medical technology may only yield survival benefit among patients with stages I–III endometrial carcinoma, but not among those with clinical stage IV disease. This study used routine clinical data to construct a nomogram model of 3- and 5-year CSS and OS among patients with endometrial carcinoma. This model provides a good prognostication tool for the clinical practice of gynecologists and general practitioners.

## Data Availability Statement

Publicly available datasets were analyzed in this study. This data can be found here: https://seer.cancer.gov/data/.

## Ethics Statement

Ethical review and approval was not required for the study on human participants in accordance with the local legislation and institutional requirements. Written informed consent for participation was not required for this study in accordance with the national legislation and the institutional requirements.

## Author Contributions

LZ: conception of the work, data collection, data analysis and interpretation, drafting the article, critical revision of the article, and final approval of the version to be published. XS and WB: conception of the work.

### Conflict of Interest

The authors declare that the research was conducted in the absence of any commercial or financial relationships that could be construed as a potential conflict of interest.

## Abbreviations

SEER: The linked Surveillance, Epidemiology, and End Results
CSS: Cancer-specific Survival
OS: Overall Survival
FIGO: The International Federation of Gynecology and Obstetics.

## References

[1] Siegel RL Miller KD Jemal A Cancer statistics, 2016. CA Cancer J Clin. (2015) 66:7–30. 10.3322/caac.21332 26742998

[2] Lewin SN Ni HTM Deutsch I Burke WM Sun X Wright JD. Comparative performance of the 2009 international federation of gynecology and obstetrics' staging system for uterine corpus cancer. Obstet Gynecol. (2010) 116:1141–9. 10.1097/AOG.0b013e3181f39849 20966700

[3] Beller U Benedet JL Creasman WT Ngan HY Quinn MA Maisonneuve P. Carcinoma of the vagina. FIGO 26th annual report on the results of treatment in gynecological cancer. Int J Gynaecol Obstet. (2006) 95:161–92. 10.1016/S0020-7292(06)60029-5 17161165

[4] Ouyang D Li R Li Y Zhu X A 7-ncRNA signature predict prognosis of Uterine corpus endometrial carcinoma. J Cell Biochem. (2019) 120:18465–77. 10.1002/jcb.29164 31168849

[5] Warren JL. Overview of the SEER-Medicare data: content, research applications, and generalizability to the United States elderly population. Med Care. (2002) 40(Suppl. 8):IV-3–18. 10.1097/00005650-200208001-00002 12187163

[6] Camp RL. X-Tile: a new bio-informatics tool for biomarker assessment and outcome-based cut-point optimization. Clin Cancer Res. (2004) 10:7252–9. 10.1158/1078-0432.CCR-04-0713 15534099

[7] Pecorelli S. Revised FIGO staging for carcinoma of the vulva, cervix, and endometrium. Int J Gynecol Obstet. (2009) 105:103–4. 10.1016/j.ijgo.2009.02.012 19367689

[8] National Comprehensive Cancer Network. (NCCN) Clinical Practice Guidelines in Oncology. Uterine Neoplasms, Version 4 (2019). Available online at: https://www.nccn.org/professionals/physician_gls/pdf/uterine.pdf

[9] Dunton CJ Pfeifer SM Braitman LE Morgan MA Carlson JA Mikuta JJ. Treatment of advanced and recurrent endometrial cancer with cisplatin, doxorubicin, and cyclophosphamide. Gynecol Oncol. (1991) 41:113–6. 10.1016/0090-8258(91)90268-A 2050302

[10] Decruze SB Green JA. Hormone therapy in advanced and recurrent endometrial cancer: a systematic review. Int J Gynecol Cancer. (2007) 17:964–78. 10.1111/j.1525-1438.2007.00897.x 17442022

[11] Abu-Rustum NR Zhou Q Gomez JD Alektiar KM Hensley ML Soslow RA. A nomogram for predicting overall survival of women with endometrial cancer following primary therapy: toward improving individualized cancer care. Gynecol Oncol. (2010) 116:399–403. 10.1016/j.ygyno.2009.11.027 20022094 PMC3870336

[12] Kosary CL. Figo stage, histology, histologic grade, age and race as prognostic factors in determining survival for cancers of the female gynecological system: an analysis of 1973-87 SEER cases of cancers of the endometrium, cervix, ovary, vulva, and vagina. Semin Surg Oncol. (1994) 10:31–46. 10.1002/ssu.2980100107 8115784

[13] Dai Y Dong Y Cheng Y Hou H Wang J Wang Z Prognostic significance of lymphovascular space invasion in patients with endometrioid endometrial cancer: a retrospective study from a single center. J Gynecol Oncol. (2019) 31:e27 10.3802/jgo.2020.31.e27 PMC7189077 31912681

[14] Ørtoft G Lausten-Thomsen L Høgdall C Hansen ES Dueholm M. Lymph-vascular space invasion (LVSI) as a strong and independent predictor for non-locoregional recurrences in endometrial cancer: a danish gynecological cancer group study. J Gynecol Oncol. (2019) 30:e84. 10.3802/jgo.2019.30.e84 31328462 PMC6658591

